# From Theory to PrACTice: A Cognitive Remediation Program Based on a Neuropsychological Model of Schizophrenia

**DOI:** 10.3389/fpsyt.2015.00169

**Published:** 2015-12-01

**Authors:** Delphine Fabre, Aurélie Vehier, Gabrielle Chesnoy-Servanin, Nicolas Gouiller, Thierry D’Amato, Mohamed Saoud

**Affiliations:** ^1^EA 4615, Centre Hospitalier le Vinatier, Université de Lyon, Bron, France; ^2^Unité Polaire de Psychoéducation (UPP), Centre Hospitalier le Vinatier, Bron, France; ^3^Centre Hospitalier le Vinatier, Bron, France

**Keywords:** schizophrenia, willful actions, cognitive control mechanism, cognitive remediation, neuropsychological model of schizophrenia

## Abstract

Cognitive dysfunction is one of the hallmark deficits of schizophrenia. A wide range of studies illustrate how it is strongly interconnected to clinical presentation and daily life functioning [see Ref. (1, 2)]. Hence, cognition is an important treatment target in schizophrenia. To address the challenge of cognitive enhancement in schizophrenia, a large number of cognitive remediation programs have been developed and evaluated over the past several decades. First, an overview of these programs is presented highlighting their specificity to cognitive deficit in schizophrenia using an integrated method. In this case, cognitive training focuses on enhancing several elementary cognitive functions considered as a prerequisite to social skills or vocational training modules. These programs are based on the neurodevelopmental hypothesis of schizophrenia. However, moderate improvement for patients who benefit from these therapies has been observed as described in Wykes et al. review (3). Next, neuropsychological models of schizophrenia are then presented. They highlight the critical role of the internally generated intentions in appropriate willful actions. The cognitive control mechanism deals with this ability. Interestingly, available cognitive remediation programs have not been influenced by these models. Hence, we propose another alternative to set up a specific cognitive remediation program for schizophrenia patients by targeting the cognitive control mechanism. We describe the PrACTice program that is in the process of being validated.

## Conceptual Frameworks Guiding Cognitive Remediation Therapies in Schizophrenia

Current cognitive remediation therapies have been developed under two main influences: the neurodevelopmental model of schizophrenia and works completed in cognitive remediation of traumatic brain injury patients. The neurodevelopmental model of schizophrenia suggests that subtle abnormalities are acquired *in utero* or perinatally and result in abnormal neurodevelopment. Subsequently, the presence of stressors when the brain maturation reaches a critical stage may interact with the weakness of certain neurological areas, resulting in cognitive dysfunctions. This line of thought is based on the vulnerability-stress model. The cognitive dysfunctions are rather global as it includes attention, processing speed, executive functioning, verbal fluency, verbal memory, and learning (4, 5). These domains have been associated with poor functional and social outcomes [e.g., Ref. (6)] as well as disorganization and negative symptoms (2). Hence, numerous intervention programs have been designed, applied, and evaluated, with the objective of improving these cognitive mechanisms in schizophrenia. Originally, cognitive therapies have also been influenced by techniques that have been developed to help brain-injured people, to recover cognitive functions. Usually, it consists of specific module training for specific cognitive deficits (attention, processing speed, executive functioning modules, etc.). For instance, the cognitive remediation therapy (CRT) (7, 8) consists of three training modules – cognitive flexibility, working memory, and planning function – rated from extremely easy to easy (the “scaffolding” technique in a drill and practice training). In the cognitive flexibility module, patients are asked to cross off the odd or even numbers inside a page with a set of numbers. It then requires to shift between odd or even numbers when the rule changes. In the memory module, individuals have to not only recall the numbers of tokens in series of lines but also transform this information by recalling the lines in different orders. Different strategies may be useful as categorizing and chunking information or self-instructional training in the use of mnemonic strategies (“drill and strategy” training). In the planning module, individuals have to plan a sequence of moves to achieve a goal. The REHA-COM^®^ program [for instance, Ref. (9)], RECOS^®^ (10) or the neurocognitive enhancement therapy [NET *only* (11)] trainings are computer assisted and also consist in different modules, which stimulate isolated cognitive mechanisms, such as memory, attentional, visuo-spatial, speed processing, or executive capacities. These programs graduate from low to higher complexity of the material on which patients are training and information-processing strategies can be recommended by the therapist. Once a participant obtains 90% accuracy at a given complexity level, the parameters of the task are changed to make the task more challenging. Among the course of studies that assess the benefits of cognitive training for schizophrenia, several cognitive remediation programs have thought to be even more specific to schizophrenia characteristics. The issues of social functioning, vocational outcomes, and motivational aspects thus became the core target of these cognitive training programs. The integrated psychological treatment (IPT) (12, 13) is the first program to be developed in this way. It is based on structured sessions, aimed to improve elementary cognitive mechanisms first and then social skills. The theoretical framework assumes that elementary neurocognitive functions are necessary prerequisites for higher order complex social functions. This program proposes sessions that include cognitive training as differentiation (i.e., abstraction and conceptual organization training) before training social perception, verbal communication, social skills, and interpersonal problem solving. Another program, the cognitive enhancement therapy [CET (14)] has been influenced in its conceptualization both by works within traumatic brain-injured patients (15) and by Brenner’s IPT program. It consists of a computer-assisted program training attention, memory, and problem-solving abilities associated with group exercises to improve social cognition. Group exercises in this latter domain consist, for instance, of solving real life social dilemmas, in abstracting themes from newspaper articles, judging affect or social contexts, initiating and maintaining conversations, etc. To target the interaction between cognitive trouble and negative symptoms, other programs associate computer-assisted cognitive training with a supportive employment program as in the thinking skills for work program (TSWP) [see Ref. (16)]. In the *NET plus* program, the NET is in combination with work therapy (11, 17). The cognitive training consists of repeated practice on computer-based exercises for attention, memory, executive function, and a dichotic listening task. A modified form of Psychological Software Services (CogReHab software) has been designed for individuals with compromised brain function. Task parameters are initially designed to be easy enough for each patient to achieve the task. For instance, sustained visual attention is trained with the visual tracking I module. The exercise begins with a black line moving across the computer screen with a red background color. As one line moves, yellow cubes appear along the line. The patient is required to focus visually at the end of the line and click the left mouse button whenever a yellow cube appears. Changing the speed of the black line’s movement or the duration of the task increases task difficulty. Patients are paid for doing cognitive exercises ($3.40/h). The work therapy consisted of payment for work activity. The work therapy packaging encompasses among others: job placement, individual counseling when problems arise, individual vocational counseling, and a certificate of participation in the program. The most common work sites are in the dietetics department, mail room, grounds, maintenance department, patient transport, and medical administration. In the neuropsychological educational approach to remediation program [NEAR, developed by Medalia and Freilich (18)], participants are referred to as learners or students, which implies that they are engaged in learning. The theoretical foundation of NEAR emphasizes motivation, social aspect of rehabilitation, such as personalization and contextualization (19). Personalization refers to the fact that the learning environment is “customized” for the participant: when a patient arrives for the rehabilitation session, he/she takes their folder and sign-in, just as they might do at work. Contextualization refers to the fact that participants have discussions on how learned skills may be relevant to everyday functions.

## An Alternative Conceptual Framework: From a Global Cognitive Deficit Hypothesis to A Single Cognitive Mechanism Deficit

**Figure 1:**
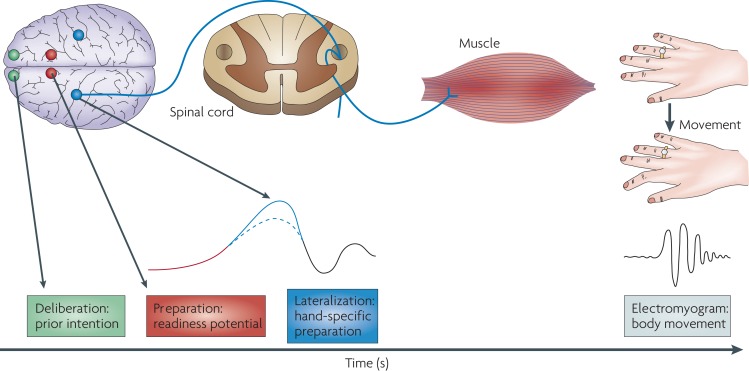
**Brain circuits for voluntary action**. Taken from Haggard (30).

As explained beforehand, most cognitive training programs that are available have been conceptualized within the hypothesis that individuals with schizophrenia suffer from a global cognitive deficit. It is suggested by the neurodevelopmental model of schizophrenia (see above). The cognitive heterogeneity characterizing schizophrenia (20) also contributes to target multiple elementary cognitive domains, in particular, attention, memory, executive functions, and social cognition deficits in a cognitive training program. Nevertheless, contemporary cognitive neuroscience provides a unifying theory for the cognitive and neural abnormalities underlying higher cognitive dysfunctions (21, 22). In this theory, an impaired cognitive control process would be expected to lead to a range of cognitive deficits across a broad range of “domains” of higher cognition. According to Lesh et al. (21), “*A piecemeal approach to cognition in schizophrenia may obscure the fact that the failure of a singular overarching cognitive domain could yield a substantial proportion of the varied pattern of cognitive deficits that are observed in the disorder*.” The cognitive control mechanism would be that mechanism dysfunctioning in schizophrenia (23). This mechanism allows guiding thought and action in accord with internal intentions and with stimulus relevant for the intended behavior [i.e., context representation in Ref. (24, 25)]. That is, it deals with neuroscience of willful action. Several neuropsychological models of willful actions in schizophrenia have been described (26–29) without influencing cognitive training program for individuals with schizophrenia. For instance, the Frith’s model states that symptoms of schizophrenia as negative and disorganized symptoms arise from diminished capacity to regulate willed (or goal directed) and stimulus-driven action systems. Indeed, from a cognitive view, the willed action pathway implies the transfer of internally generated goals and intentions into a reliable action response. By contrast, the stimulus-driven action pathway mediates actions that are triggered by stimulus environment. As actions driven by the current stimulus environment may be incompatible with an individual’s goals, the regulation or control of these distinct action pathways is necessary to act in a goal-directed manner. From a neuronal view, two distinct action pathways are well known. The specific brain circuits for voluntary action are of particular interest in Frith’s model. It implies the primary motor cortex (M1), which receives information from the supplementary motor area (SMA) and the presupplementary motor area (preSMA), which in turn receives inputs from the basal ganglia and the prefrontal cortex. Hence, the brain activity preceding a voluntary action of the right-hand proceeds as follows: the frontopolar cortex (shown in green) forms and deliberates long-range plans and intentions. The preSMA (shown in red) begins the preparation of the action; together with other premotor areas, it generates the readiness potentials (RPs) (red trace) that can be recorded from the scalp. Immediately before the action takes place, M1 (shown in blue) becomes active (Figure 1).

That is, to achieve a goal, an individual must activate the voluntary action pathway who simultaneously suppresses the stimulus-driven pathway (28). The inability to appropriately activate the willed action pathway can lead to a decrease in goal-directed behaviors consistent with the negative symptoms observed in schizophrenia. Another consequence of poor regulation of the two action pathways may be fragmented, disorganized behavioral output. This poor regulation consists of a neurophysiological disturbance in the efference copy that indexes the willed or intentional nature of human action (26).

In the model developed by Jeannerod and collaborators (31), the focus is on the delusion symptom in schizophrenia. Patients suffering from delusions of control may report that their movements are triggered by someone else: the sense of agency is disrupted. To explain such symptomatology, the theoretical field also deals with the two distinct pathways underlying willful actions and stimulus-driven actions. From the activation of the voluntary circuit, two distinct subjective experiences arise: the experience of intention and the experience of agency (32). Empirically, the experience of agency has been shown in studies with healthy individuals. In Fourneret and Jeannerod (33), healthy participants were requested to carry out simple finger and wrist movements. No direct visual control of their hand was available. Instead, the video capture of either their own hand or an alien hand executing the same or a different movement was displayed on a screen in real time. The task was to answer whether the hand displayed on the screen was their own or not. Healthy participants easily performed the task despite the experimental design. The removal of direct sensory feedback did not interfere with their ability to discriminate their own hand from the alien one. It suggests that the sense of agency depends on early processes rather than late stage processes while a subject is executing a volunteer movement. The sensory feedback from the action itself is not to a large extent relevant for the experience of agency. More precisely, the mechanism that explains how early processes in action lead to the sense of agency is related with the forward comparator. This neurological mechanism, involved in volunteer action, compares efference copy of motor commands with the sensory feedback issued from the movement. The experience of intention, which initiates action, can be highlighted using Libet’s experimental design (34). It consists of participants in watching a spot rotating every 2560 ms on a screen. At a time of their own choosing, participants make a voluntary movement of the right-hand. A random time later, the rotating spot stops and participants have to indicate where the spot was when they first felt the urge to move. This is the time of “conscious will.” The average time of this judgment was 206 ms before the onset of muscle activity. During this experiment, Libet and collaborators also measured the preparatory brain activity preceding voluntary action, what he calls the RP. He observed that the RP onset preceded will judgment by several hundred milliseconds. These outcomes show that an unconscious neural process initiates action and produces the conscious experience of intention.

In a neuropsychological model of willful action, we can also state the viewpoint defended in Lafargue et al. (35). It is of particular interest to account for schizophrenia symptomatology, especially the subjective experience of lack of control as a low degree of delusions of alien control. When a patient reports passivity experiences, in most cases, he does not merely report a lack of agency. He reports instead a simple feeling of a lack of control of his own acts. A continuum stretching between full control of one’s own acts and the lack of agency may exist. The *sense of effort mechanism* (36) would be the core mechanism of this continuum. Paretic patients could characterize full control of their own acts at one end of a continuum: when they attempt to move their paretic limb, these patients report that they feel as if they were producing a considerable amount of force against a resistance. At the other end of the continuum, schizophrenia patients who describe delusions of alien control. When they act, they report, for instance, “They inserted a computer in my brain” and “it makes me turn to the left or right” [Ref. (37), p. 358]. Hence, according to Lafargue et al. (35), the sense of effort is at fault in schizophrenic patients with passivity experiences. Patients should encounter difficulties when monitoring intended effort or when appreciating achieved effort. They supported this hypothesis with a particular task that consists of asking participants to produce muscular contractions of different intensities in response to numerical values given by the experimenter: 1, 3, 5, 7, or 9 on a 10-points scale, “0”: no effort; “10”: maximum effort. Schizophrenia and control participants were able to maintain a linear and statistically significant relationship between the numerical values and the exerted forces. However, the strength of this relationship was much weaker, on average, for both groups of patients with schizophrenia. It was particularly poor for the group with schizophrenic patients experiencing passivity.

With the PrACTice program, we attempt to rehabilitate the cognitive component that is prominent to initiate willful actions. It refers to the *cognitive control mechanism* (21, 22, 24). In neuropsychological models of schizophrenia, it concerns the mental representations underlying plan or intention. The rational is as follows: a robust mental representation of our internally generated intentions would increase the opportunity to initiate a reliable action. Metaphorically, PrACTice aims at taking on the orange traffic light function when it precedes the green light. Namely, it allows initiating the action to move more efficiently. The orange light signal in PrACTice is the generation of mental representations of a goal or intention related to a related objective.

## Description of the PrACTice Program

The PrACTice cognitive training consists of 24 sessions with the PrACTice software program (4 sessions per week during 6 weeks) associated with homework between each session. The PrACTice program includes 12 training lists distributed into 3 domains – Daily life; Bodily Hygiene and Life Hygiene; and Administrative Work and Travel – and 4 difficulty levels. During one session, the participants perform two lists among the three domains and for one difficulty level. Each list contains 20 trials. Each trial begins with a goal-directed action sentence displayed in the center of a computer screen for 4000 ms (e.g., “writing a letter”). A scene containing contextual information is then displayed for 4000 ms, followed by the picture of an isolated object. In a maximum time of 4000 ms, subjects have to answer whether the object is useful or not to achieve the goal-directed action. For one trial, there are six pairs of prime-target pictures in a row. There are three useful targets and three non-useful targets. Targets are randomized inside each trial. However, each trial is always displayed in the same order. In level 1–3, trials begin with 1–3 goal-directed action visually displayed, respectively. In level 4, only one goal-directed action is displayed, but no contextual information is displayed. Participants perform each list of each difficulty level twice (24 sessions).

During the training sessions, participants have to imagine performing the goal-directed action. Pictures with contextual information are displayed to help them: in a bubble speech, they have instructions such as “Which tools would be useful to achieve the objective?” and “How will you do it?” (see Figure 3). In levels 2 and 3, as there are several objectives, contextual information-guided participants to imagine achieving the right objective. In level 4, participants have to imagine the context and how they will achieve objectives. The accuracy and time of response are recorded as a measure of the effort made to produce a mental representation of the action. Indeed, decision times informed us on the process confronting the target that is displayed and the activated mental representations associated with the task to imagine the action and tools useful to achieve objectives. It allows a measure of the effort made to produce a mental representation of actions relative to the different objectives proposed. Furthermore, the PrACTice program includes 2 short-lists containing 10 trials (10 objectives) that are not used for training during the 24 sessions. These two lists include identical objectives, contexts, and targets but one list does not display the strategy to imagine how to achieve the objectives displayed by the program. These lists are used to introduce the training sessions with the program and is used as pre and post evaluation in the process of validation of the program.

**Figure 2:**
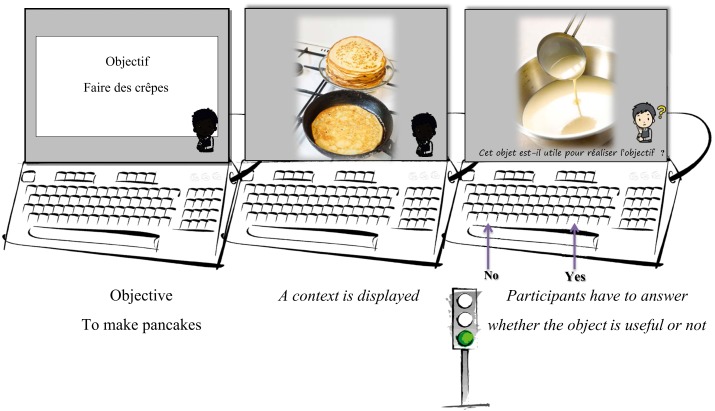
**Illustration from the inactive short-list, with the trial “To make pancakes” as objective**. No strategy is suggested to make the decision task. The target required a “useful” answer.

**Figure 3:**
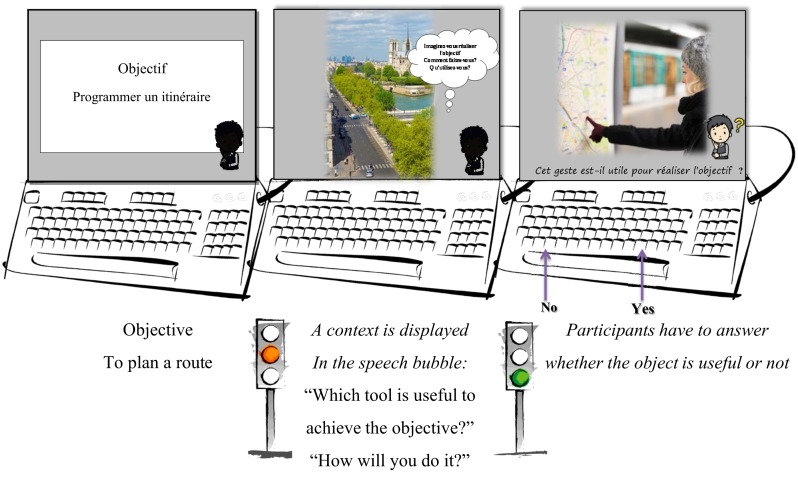
**Illustration from the active short-list, with the trial “To plan a route” as objective**. The strategy is suggested in the speech bubble before to make the decision task. “Which tools would be useful to achieve the objective?” “How would you do it?” The target required a “useful” answer.

### Procedure for Each Participant

#### Psychoeducation Sessions

Psychoeducation sessions (two sessions) are proposed on cognitive mechanisms at the beginning of the program. These sessions can be individual or in group. The objective for the patients is to understand that behavior and decision-making are done thanks to several cognitive mechanisms. Functions of attention, memory, and perception are then discussed, including their utility in daily life. A picture is used to illustrate a situation in which a character does not trigger certain cognitive mechanisms during an activity. Next, the questions concerning actions that are stimulus induced or externally caused and actions that are voluntary or self-initiated are discussed. It brings the role of our goal and intention to initiate some of our actions. Then, metaphorical language is used to clarify how to strengthen the internal representation, which inaugurates voluntary actions. The green light allows drivers to go through the intersection. The red signal prohibits any traffic from proceeding. The orange light provides warning that the signal will be changing from green to red or from red to green in certain countries, such as in the UK. We explain that usually, when the red traffic light turns green, an externally caused action is expected: to move forward or to go through the intersection. When the red traffic light turns orange it signals that in a few seconds it will turn green. When the orange light is displayed drivers should prepare to set off. The action will be then more self-initiated. Usually, drivers lose less time to set off when the orange traffic light is displayed before turning green. Finally, individuals have to observe in which daily life circumstances they could be in the same frame of mind as a driver when the orange light is displayed (after the green light). They have to identify several personal objectives.

Participants also experiment the inactive PrACTice short-list (see Figure 2) that is similar to the training lists but include only 10 trials, with objectives that will not be trained during the training sessions and which do not proposed the coaching strategies.

When 10 objectives are completed, the participant talks about his/her experience. The second short-list is then proposed with a strategy coaching approach (the Active Short-list): participants have to imagine performing the goal-directed action when pictures with contextual information are displayed. To help them, instructions such as “which tools would be useful to achieve the objective?” or “how would you do it?” are written in a speech bubble (see Figure 3).

Then, we discuss about the accuracy and time of response recorded as a measure of the effort made to produce a mental representation of the action. If the goal-directed actions are mentally represented, patients gradually need less time to answer whether an object is useful or not.

#### Training Sessions

Participants began on level 1. In one session, they performed two training lists among the three domains (Daily life; Bodily Hygiene and Life Hygiene; and Administrative Work and Travel) and with the strategy to imagine achieving the objectives displayed by the program. For instance, in the Daily life domain objectives such as “To vacuum the living room” or “To take out the trash” are displayed. In Hygiene and Life Hygiene task (two separate lists have been constructed for men and women) objectives such as “To provide a balanced meal” or “To take a shower” are displayed. In Administrative Work and Travel, objectives such as “To validate a public transportation ticket” or “To sort out regular mail” are displayed. When each training list on level one is performed twice, participants are trained on level 2. Trials begin with two goal-directed actions visually displayed, respectively. Hence, when contextual information is displayed, participants have to be particularly mindful since it determines which objective they have to imagine completing. When each training list on level two is performed twice, participants are trained on level 3. Each trial in this level begins with three goal-directed actions visually displayed, respectively, in a logic sequence: “To wash the salad,” “To drain the salad,” and “To serve with salad dressing.” When each training list on level three is performed twice, participants are trained on level 4. In level fou4, only one goal-directed action is displayed, but no contextual information is displayed. Participants have to imagine the context and how they would complete the objective.

#### Homework

Throughout the training sessions, participants have to apply the strategy to imagine completing at home their objectives identified with the therapist. An agenda is filled by participants between each session. They point out how many times they used the strategy with the related objective.

Hence, PrACTice is a drill and strategy coaching approach cognitive remediation program. The cognitive mechanism targeted is specific to schizophrenia disease. The cognitive control mechanism is at the core of the program. To facilitate generalization of cognitive gains to everyday life, concrete goals are defined according to the patient’s interests. Moreover, during the course of the therapy, participants fill in an agenda to indicate how many times they use the strategy at home to promote its use in daily life.

## Discussion

The elementary role of internally generated intentions in appropriate actions and the cognitive control mechanism is at core of the PrACTice program. Substantial benefits for patients who will benefit from this program are then expected, in regards of their negative symptomatology as well as disorganization symptomatology. A generalization of these benefits in daily life functioning is also expected. Presently, the principal limitation of the program comes from the absence of strategy if interference with emotion occurs while patients are carrying out an activity in daily life. It can limit their capacity to generate intention as trained with the PrACTice program. It is well known that emotions such as anxiety decrease cognitive resources while the cognitive control mechanism is effortful for patients. For this purpose, psychotherapy sessions, training emotion regulation, could be associated with the PrACTice program. Moreover, it should strengthen the ecological value of the program.

Interestingly, impairments of generation of intentions and appropriate actions are operating not only in schizophrenia but also in other psychotic disorders. In particular, these mechanisms can be impaired in patients with depression or obsessive–­compulsive disease (OCD). In depression, the cognitive control mechanism is most certainly impaired for melancholic states, like in schizophrenia: the cognitive control mechanism is “out of order” (failed) leading to decreased generation of intention and therefore behavior with abnormalities of volition is encountered. According to Damasio and Damasio (38), these psychopathic behaviors pertain to what they called the “sick-will.” For major depression, the cognitive control mechanism is mostly dedicated to negative thought and action in accordance to sad intention and could lead to initiate desperate actions. To a certain degree, it can be supposed to be similar in OCD: the cognitive control mechanism is “in order,” but this time mostly dedicated to obsessive thoughts. The theoretical framework of Cohen et al. (24) states that the control mechanism allows guiding thought and action in accord with internal intentions and with relevant stimulus for the intended behavior (i.e., context representation). It can be hypothesized that obsessive thought is related to the relevant stimulus for compulsions that are related to the intended behavior. Hence, the mechanism initially dedicated to favor willful acts is abused. Further versions of the PrACTice program could be set up to be specific to these latter diseases.

## Conflict of Interest Statement

The authors declare that the research was conducted in the absence of any commercial or financial relationships that could be construed as a potential conflict of interest.
